# Targeting the m6A mRNA demethylase FTO suppresses vascular endothelial growth factor release and choroidal neovascularization

**DOI:** 10.1038/s41392-022-01277-4

**Published:** 2023-02-20

**Authors:** Shao-bin Wang, Yosuke Nagasaka, Dionne Argyle, Ayami Nagasaka, Praveen Yerramothu, Bradley D. Gelfand, Jayakrishna Ambati

**Affiliations:** 1 Center for Advanced Vision Science, University of Virginia School of Medicine, Charlottesville, VA USA; 2 Department of Ophthalmology, University of Virginia School of Medicine, Charlottesville, VA USA; 3 Department of Biomedical Engineering, University of Virginia School of Medicine, Charlottesville, VA USA; 4 Department of Pathology, University of Virginia School of Medicine, Charlottesville, VA USA; 5 Department of Microbiology, Immunology, and Cancer Biology, University of Virginia School of Medicine, Charlottesville, VA USA

**Keywords:** Epigenetics, Oculomotor system

**Dear Editor**,

Vascular endothelial growth factor-A (VEGFA, also known as VEGF) is a critical angiogenic factor that regulates the physiological and pathological blood vessel growth.^1^ Increased abundance of VEGF in the eye underlies many forms of aberrant ocular angiogenesis and resultant vision loss, including in neovascular age-related macular degeneration (nvAMD), proliferative diabetic retinopathy (PDR), ischemic retinal vein occlusion, and retinopathy of prematurity (ROP). Multiple VEGF inhibitors are approved for such ocular angiogenic diseases. Despite the initial, and often dramatic, efficacy of anti-VEGF therapy, real-world and long-term studies are more sobering.^2,3^ Thus, enhanced understanding about the regulation of ocular VEGF can further elucidate the underlying pathological mechanisms and aid in developing new therapeutic strategies.

N6-methyladenosine (m6A), the most abundant post-transcriptional modification of eukaryotic mRNA, plays fundamental roles in regulating biological processes and diseases.^4^ The m6A modification is dynamic, being “written” by methyltransferase complex components, and “erased” by demethylases, including Fat mass and Obesity-associated protein (FTO) and AlkB Homolog 5, RNA Demethylase (ALKBH5). The best studied effect of the m6A modification is promotion of mRNA instability, thereby affecting target mRNA transcript abundance. Accordingly, m6A RNA modifications are essential for macrophage activation,^5^ which was found to be crucial for the development of experimental nvAMD in our prior studies.^6^ However, whether m6A modification of macrophage genes plays a role in nvAMD is unknown.

We studied the role of the m6A methyltranscriptome in laser photocoagulation-induced choroidal neovascularization. We observed an increased abundance of *Vegfa* mRNA in angiogenic choroid, accompanied by enhanced levels of *Fto* mRNA (Fig. 1a) and modestly decreased levels of *Rbm15* and *Wtap* mRNAs (Supplementary Fig. S1a). However, there were no significant changes in *Metttl3*, *Mettl14*, and *Alkbh5* mRNA abundance (Fig. 1a and Supplementary Fig. S1a). At 3 days after laser injury, coinciding with macrophage infiltration and the onset of neovascularization,^7^ we observed a dramatic increase in FTO-expressing cells within the area of neovascularization, some but not all of which were F4/80^+^ (Fig. 1b, c, d, e and Supplementary Fig. S1b, c). Inhibiting FTO activity in vivo using a selective inhibitor resulted in a significant reduction in neovascularization but, interestingly, not in F4/80+ macrophage recruitment (Fig. 1f, g, h). In addition, inhibition of FTO suppressed VEGFA protein levels in laser treated RPE-choroid tissue (Fig. 1i) and suppressed the VEGFA release in human ARPE-19 cells (Supplementary Fig. S1d). In primary mouse bone marrow derived macrophages, knockdown of FTO expression by specific small interfering RNA (siRNA) significantly dampened macrophage-mediated VEGFA release (Supplementary Fig. S1e, f, g).

**Fig. 1:**
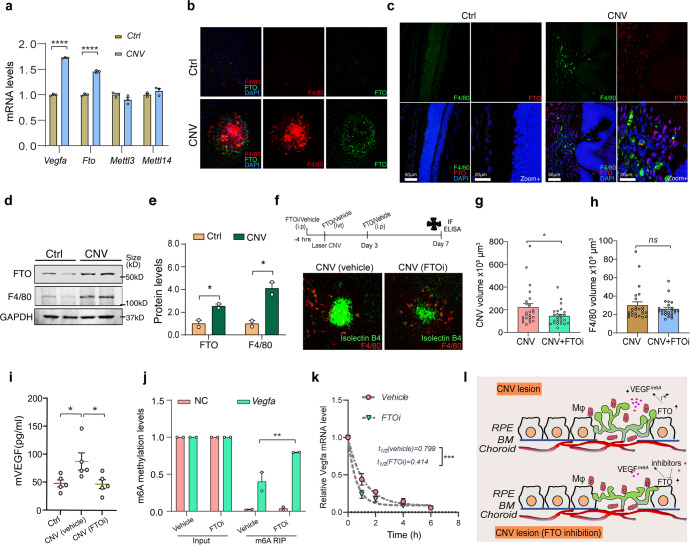
FTO in macrophage VEGFA release and choroidal neovascularization. **a** Quantification of *Vegfa*, m6A methyltransferase (*Mettl3, Mettl4*), demethylase (*Fto*) mRNA levels in pooled eye tissues (*n* = 3) of control, naive (no laser treated) mice (Ctrl) or mice following laser injury (choroidal neovascularization, CNV, day 3 after laser injury). **b**, **c** Immunofluorescent staining of FTO in flat-mounted RPE-choroid tissues (**b**) and cryosections of eyes (**c**) at 3 days after laser injury. F4/80 immunostaining indicates macrophage infiltration following laser injury. **d**, **e** Immunoblotting and quantification of FTO and F4/80 protein levels in RPE-choroid tissues isolated from mice eyes at 3 days after laser injury (*n* = 2 eyes). **f** Immunofluorescent staining of neovascularization using isolectin B4 (green) and of macrophages by F4/80 (red) in RPE-choroid tissues of mice treated with FTO inhibitor (FTOi) or vehicle, at 7 days after laser injury. **g**, **h** Quantification of CNV and F4/80 volumes based on isolectin B4 and F4/80 staining in RPE-choroid tissues of mice treated with FTO inhibitor (FTOi) or vehicle, at 7 days after laser injury (*n* = 24 laser spots for Ctrl, and n = 25 spots for FTOi). **i** Quantification of VEGFA levels in the RPE/choroid tissues of mice treated with FTO inhibitor (FTOi) or vehicle, at 3 days after laser injury (*n* = 5 eyes). **j** Quantification of methylated *Vegfa* mRNA levels in mouse BMDMs treated with FTO inhibitor (FTOi) or vehicle for 24 h by using MeRIP-qPCR (*n* = 2). **k** Determination of *Vegfa* mRNA stability in BMDMs pretreated with FTO inhibitor (FTOi) or vehicle, followed with Actinomycin D inhibition (10 µg/ml). mRNA abundance was measured by RT-qPCR at the indicated time points and *Vegfa* mRNA half-lives (*t*_1/2_) determined by fitting the data to a nonlinear one phase decay model (mean ± SEM, *n* = 3). **l** Schematic diagram showing FTO regulates VEGFA release and choroidal neovascularization in AMD. Retinal pigment epithelium RPE, BM Bruch’s membrane, Mφ macrophages. Data are shown as mean ± SEM, *p < 0.05; ** *p* < 0.01; ***p < 0.001; ****p < 0.0001; *ns* not significant. Two-way ANOVA with Sidak’s multiple comparisons (**a**, **c**); One-way analysis of variance (ANOVA) with Dunnett’s multiple comparisons (**g**); unpaired two-tailed *t*-test (**e**, **j**, **g**, **k**)

Next, we used Methylated RNA ImmunoPrecipitation PCR (MeRIP-PCR) to determine whether FTO demethylated macrophage *Vegfa* mRNA. Consistent with previous studies,^8^ we found *Vegfa* mRNA is abundantly methylated in basal conditions (Fig. 1j). Inhibition of FTO significantly increased the abundance of m6A methylated *Vegfa* mRNA; this was accompanied by a reduction in mouse VEGFA release (Fig. 1i and Supplementary Fig. S2a to c). In contrast, targeting m6A methylase with a METTL3 inhibitor resulted in a dose-dependent increase in VEGFA release (Supplementary Fig. S2d). FTO regulates gene expression via maintaining mRNA stability.^9^ After blocking new mRNA synthesis, we found that macrophage *Vegfa* mRNA half-life was significantly shorter in the presence of an FTO inhibitor (Fig. 1k and Supplementary Fig. S2e). However, FTO inhibition did not significantly alter the mRNA abundance of other pro-angiogenic factors, such as placental growth factor (*Plgf*) and platelet-derived growth factors (*Pdgfa*), suggesting the preferential effect of FTO-mediated m6A demethylation on maintaining macrophage *Vegfa* mRNA stability and VEGFA release (Supplementary Fig. S2f).

Although we found *Vegfa* mRNA was the principal pro-angiogenic RNA substrate regulated by FTO in murine macrophages, other genes could also be impacted by FTO inhibition during neovascularization. For example, FTO regulates focal adhesion kinase (FAK) expression in corneal neovascularization.^10^ Our study suggests FTO inhibition has minimal adverse effects on cell viability, and we did not observe in vivo retinal toxicity with FTO inhibition in our studies. However, more detailed toxicity studies remain to be performed. Additionally, macrophage- targeted delivery systems could be a promising approach for targeting FTO in ocular angiogenic disorders.

Collectively, our study identifies a previously undescribed role of FTO regulation of VEGFA expression and choroidal neovascularization in vivo (Fig. 1l). This work reveals a new mechanism of *Vegfa* mRNA modification that is regulated by the m6A methyltranscriptome. The discovery that inhibition of FTO suppresses VEGFA release and choroidal neovascularization opens the possibility of therapeutic targeting of FTO for angiogenic eye diseases.

## Supplementary information

Supplemental Material

Supplemental Figure 1

Supplemental Figure 2

Supplemental Figure 3

## Data Availability

All materials are available in the main text or supplementary materials. Further information and requests for resources and reagents are available from the corresponding authors on reasonable request.
