# Supplementary material for: Targeting the m6A mRNA demethylase FTO suppresses vascular endothelial growth factor release and choroidal neovascularization
Source: Signal Transduct Target Ther. 2023 Feb 20;8:72. doi: 10.1038/s41392-022-01277-4 (PMC9939410; doi:10.1038/s41392-022-01277-4)
Supplement: Supplementary file 1 — Supplemental Material [file 41392_2022_1277_MOESM1_ESM.docx]

Supplementary Materials for

Targeting the m6A mRNA demethylase FTO suppresses vascular endothelial growth factor release and choroidal neovascularization

Shao-bin Wang^1, 2*†^, Yosuke Nagasaka^1, 2†^, Dionne Argyle^1, 2^, Ayami Nagasaka^1, 2^, Praveen Yerramothu^1, 2^, Bradley D. Gelfand^1, 2, 4^, Jayakrishna Ambati^1, 2, 3, 5*^

Correspondence to: [ja9qr@virginia.edu](mailto:ja9qr@virginia.edu) or sw3ta@virginia.edu

**This PDF file includes:**

Materials and Methods

Figures S1 to S3

Supplemental References

Materials and Methods

Animals

C57BL/6 (wild type) mice were purchased from The Jackson Laboratory (Bar Harbor, ME, USA). All mice were housed in a pathogen-free laminar flow facility at the University of Virginia. For all procedures, anesthesia was achieved by intraperitoneal injection of ketamine hydrochloride (100 mg/kg; Fort Dodge Animal Health) and xylazine (10 mg/kg; Phoenix Scientific), and pupils were dilated with topical 1% tropicamide and 2.5% phenylephrine hydrochloride (Alcon Laboratories). Mice were treated in accordance with the guidelines of the University of Virginia’s Institutional Animal Care and Use Committee and the Association for Research in Vision and Ophthalmology. Both male and female mice between 6 and 8 weeks of age were used.

Laser-Induced CNV

Laser photocoagulation (532 nm, 180 mW, 100 ms, 75 μm) (OcuLight GL; IRIDEX Corp., Mountain View, CA, USA) was performed bilaterally (volume studies: 4 spots per eye; RNA analyses: 10 spots per eye) in 6- to 8-week-old mice on day 0 to induce CNV as previously described ^1-3^. For drug treatment, the mice pretreated with FTO inhibitor (FB23-2, SML2694, Sigma) or vehicle (DMSO) through intraperitoneal injection (i.p, 2mg/kg). Then, the mice were subjected to laser photocoagulation followed by intravitreous injection of the FTO inhibitor (25 ng/0.5 μl per eye) or vehicle. At day 3 after laser injury, the mice were administered the FTO inhibitor (2mg/kg) or vehicle via intraperitoneal injection. At day 7, the eyes were collected for fluorescein labeled-isolectin B4 and F4/80 staining and for neovascular volume quantification.

Cell culture

Primary mouse BMDMs were isolated as previously described ^4^. BMDMs were cultured in IMDM (Gibco) basal medium containing 10% fetal bovine serum (FBS), 30% L929 supernatants, nonessential amino acids, sodium pyruvate, 2-mercaptoethanol, and antibiotics. THP-1 cells were cultured in RPMI 1640 medium with 10% FBS and antibiotics. ARPE-19 cells were cultured in DMEM medium with 10% FBS and antibiotics. All cells were maintained at 37°C in a 5% CO_2_ environment.

Immunoblotting

Cells and tissues were homogenized in RIPA buffer (R0278, Sigma) with protease and phosphatase inhibitors (A32959, Thermo Scientific). Protein concentration was determined with a Pierce BCA Protein Assay Kit (23225, Thermo Scientific). Equal quantities of protein boiled in LDS Sample Buffer (NP0007, Thermo Scientific) were resolved by Novex Tris-glycine gels (Invitrogen) and transferred onto LF PVDF membranes (1704274, Bio-Rad). The transferred membranes were blocked with Blocking Buffer (927-40000, LI-COR) and incubated with the following primary antibodies: anti-FTO pAb (AdipoGen Life Sciences, AG-25A-0089-C100, USA), anti-GAPDH mAb (Cell Signaling Technology, 97166), anti-F4/80 (Santa Cruz Biotechnology, sc-25830). The signal was visualized with species-specific secondary antibodies conjugated with IRDye and Odyssey® CLx Imaging System.

Real-time polymerase chain reaction (RT-PCR)

Total RNA was extracted from mouse BMDMs or eye tissues with TRIzol™ Reagent (Thermo Fisher Scientific, 15596018, USA) and quantified by using a NanoDrop 2000 spectrophotometer (Thermo-Fisher Scientific, USA). Reverse transcription was performed using the QuantiTect Reverse Transcription kit (205313, QIAGEN). Target genes were amplified by real-time quantitative polymerase chain reaction (PCR) (Applied Biosystems, 7900 HT Fast Real-Time PCR system) with Power SYBR Green Master Mix. Relative gene expression was determined by the *2^−ΔΔCt^* method, and 18S rRNA or GAPDH (glyceraldehyde-3-phosphate dehydrogenase) was used as an internal control. The primers are listed in the Table below:

| Target name | Forward sequence | Reverse sequence |
| --- | --- | --- |
| *Mettl14* | TGCTCCAAACTCAAAACGGAA | ATCATTATGGGGATTTAAGCTCT |
| *Mettl3* | ATTGTTGAAAAGTTTCGCTCT | GAACATATTTGCAGGTGTCCA |
| *Rbm15* | AGCACTCTAAGCCTCAACACA | ATTTAAAAGGCAACCGGAGCAT |
| *Fto* | ACAGCCTCGGTTTAGTTCCAC | TGCCTCAGCCACTCAAACTCC |
| *Wtap* | ACAATCACGTCATAGCTACCAG | TGAGACCACACCTTCACGTT |
| *Alkbh5* | CGCGGTCATCAACGACTACCAG | AAAGCACAGGTTCCGACACCC |
| *Vegfa* | CACGACAGAAGGAGAGCAGAAG | GCTACTGCCGTCCGATTGAG |
| *Fgfb* | GAAGAGCGACCCACACGTCA | CCAGTTCGTTTCAGTGCCACA |
| *Pdgfa* | CCTTCCTGATGGCCTTACGTG | AAAATAACCGTCTCGCTGT |
| *Plgf* | CTAGAACCTGCCCTGATTCCC | AACCCAGACTTGTATCGGTCA |
| *Gapdh* | CAGGTTGTCTCCTGCGACTT | TATGGGGGTCTGGGATGGAA |
| *18S* | TTCGTATTGCGCCGCTAGA | CTTTCGCTCTGGTCCGTCTT |

RPE flat mounts and immunofluorescent staining

RPE flat mounts were prepared as previously described ^5^. Briefly, at day 3 or 7 after laser injury, mice eyes were enucleated and dissected as retina and sclera-RPE-choroid complexes. The RPE-choroid sheets were immediately fixed in 4% paraformaldehyde (Electron Microscopy Sciences, 50-980-495, USA) in phosphate-buffered saline for 1 hour. Next, the RPE-choroid sheets were permeabilized and blocked with blocking buffer (3% of Normal goat serum, 0.1% Triton-X 100 in PBA). As described previously ^1^, CNV volumes were determined by labeling with fluorescein labeled Griffonia Simplicifolia Lectin I (GSL I) isolectin B4 (Vector Laboratories, FL-1201, USA). RPE-conjugated F4/80 antibody (Bio-Rad, MCA497PET, USA) immunolabeling was used to identify infiltrating macrophages. FTO expression was visualized by immunolabeling with anti-FTO antibody (AdipoGen Life Sciences, AG-20A-0083, USA), and the specificity of labeling was assessed using isotype serum immunolabeling. The sclera-choroid/RPE complexes were flat mounted with ProLong® Gold Antifade Mountant with DAPI (Fisher Scientific, P36935, USA). Cryo-sections of 4% Paraformaldehyde (Electron Microscopy Sciences, 15714S, USA) fixed eyes were immune-labeled with anti-FTO pAb (AdipoGen Life Sciences, AG-25A-0089-C100, USA), anti-F4/80 (Bio-Rad, MCA497RT, USA) and DAPI. Fluorescence images were captured by Nikon A1R laser scanning confocal microscope.

VEGFA ELISA

The RPE-choroid complex was isolated from mouse eyes on day 3 after laser injury and treatment with FTO inhibitor or vehicle. Eye tissues were lysed by sonication with immunoprecipitation assay (RIPA) buffer (Sigma-Aldrich, R0278, USA) containing protease inhibitor (Sigma-Aldrich, 11836170001, USA), on ice for 15 minutes. The lysate was centrifuged at 20,000×g for 15 minutes at 4 °C, and total protein in the lysates was quantified using the Thermo Scientific™ Pierce™ BCA™ Protein Assay (Fisher Scientific, PI23225, USA). VEGFA protein levels in the supernatants were measured by VEGF DuoSet ELISA (R&D Systems, DY493, USA), following the manufacturer’s instructions. Mouse BMDMs or human THP-1 cells (5x10^5^ cells per well) were plated into six-well plates in complete medium and allowed to adhere overnight. Cells were then incubated for 24 hours in the absence or presence of various doses of FTO inhibitor or METTL3 inhibitors (STM2457, Selleck Chemicals). BMDMs were incubated with 125 ng/ml of LPS from Escherichia coli O111:B4 (Sigma-Aldrich, L2630, USA) for 4 hours, followed by FTO inhibitor treatment as mentioned above. The siRNAs (si*Fto*: 5'-P.G.A.G.G.A.U.C.C.A.A.G.G.C.A.A.A.G.A.U.dT.dT 3'; siCtrl: 5'-P.U.A.A.G.G.C.U.A.U.G.A.A.G.A.G.A.U.dT.dT 3'), were designed and synthesized by GE Healthcare Dharmacon, were transfected twice with BMDMs by using DharmaFECT 4 Transfection Reagent (Dharmacon, T-2004, USA). VEGFA levels in the supernatants were measured by mouse or human VEGF DuoSet ELISA (R&D Systems, DY493 or DY293B, USA), following the manufacturer’s instructions.

mRNA stability assay

Macrophage *Vegfa* mRNA stability was determined by using Actinomycin D-induced transcription inhibition as previously described ^6^. Briefly, 1x10^5^ BMDM cells per well were seeded in a 12-well plate. After adhering to culture well, the BMDMs were pretreated with DMSO or 5 µM FB23-2 (SML2694, Sigma, USA) to prevent the FTO-mediated mRNA demethylation. After 24 hours, the first well of BMDM were collected as first-time point (t=0 hour) using a cell scraper (NC1890482, Fisher Scientific, USA). The remaining wells were treated with Actinomycin D (10 µg/ml, A9415, Sigma, USA) to inhibit transcription, and cells collected at 1, 2, 4, and 6 hours after Actinomycin D inhibition. The collected cell pellets were subjected to RNA extraction using TRIzol reagent. The *Vegfa* mRNA was quantified by RT-PCR as described above.

Methylated RNA immunoprecipitation-qPCR (MeRIP-qPCR)

To validate m6A methylation of Vegfa mRNA and perturbations induced by FTOi treatment, we performed MeRIP-qPCR by following the EpiMark® N6-Methyladenosine Enrichment Kit protocol (New England Biolabs, E1610S, USA). Briefly, total RNA was extracted from mouse BMDMs treated with 5 µM of FTO inhibitor or vehicle for 24 hours by using TRIzol™ Reagent (Thermo Fisher Scientific, 15596018, USA). 100 μg of total RNA from each group was mixed with unmodified RNA (Negative control, NC). Next, 20 μg of these mixtures was incubated with m6A antibody (Cell Signaling Technology, D9D9W, USA) and then pulled down using Pierce™ Protein A/G Magnetic Beads (Thermo Fisher Scientific, 88802, USA) overnight. The enriched m6A positive RNAs were then eluted and purified by NucleoSpin RNA Clean-Up (Macherey-Nagel, 740948.25, USA), and the amounts of m6A-modified *Vegfa* mRNA or negative control RNAs were determined by reverse transcription and qPCR.

Statistics

Statistical analyses were performed using GraphPad Prism 8.0. Data were expressed as mean ± SEM and were analyzed with unpaired two-tailed *t* test, one-way analysis of variance (ANOVA) with Dunnett’s multiple comparisons, or two-way ANOVA with Sidak’s multiple comparisons tests. *P* < 0.05 was deemed statistically significant.

Figure S1


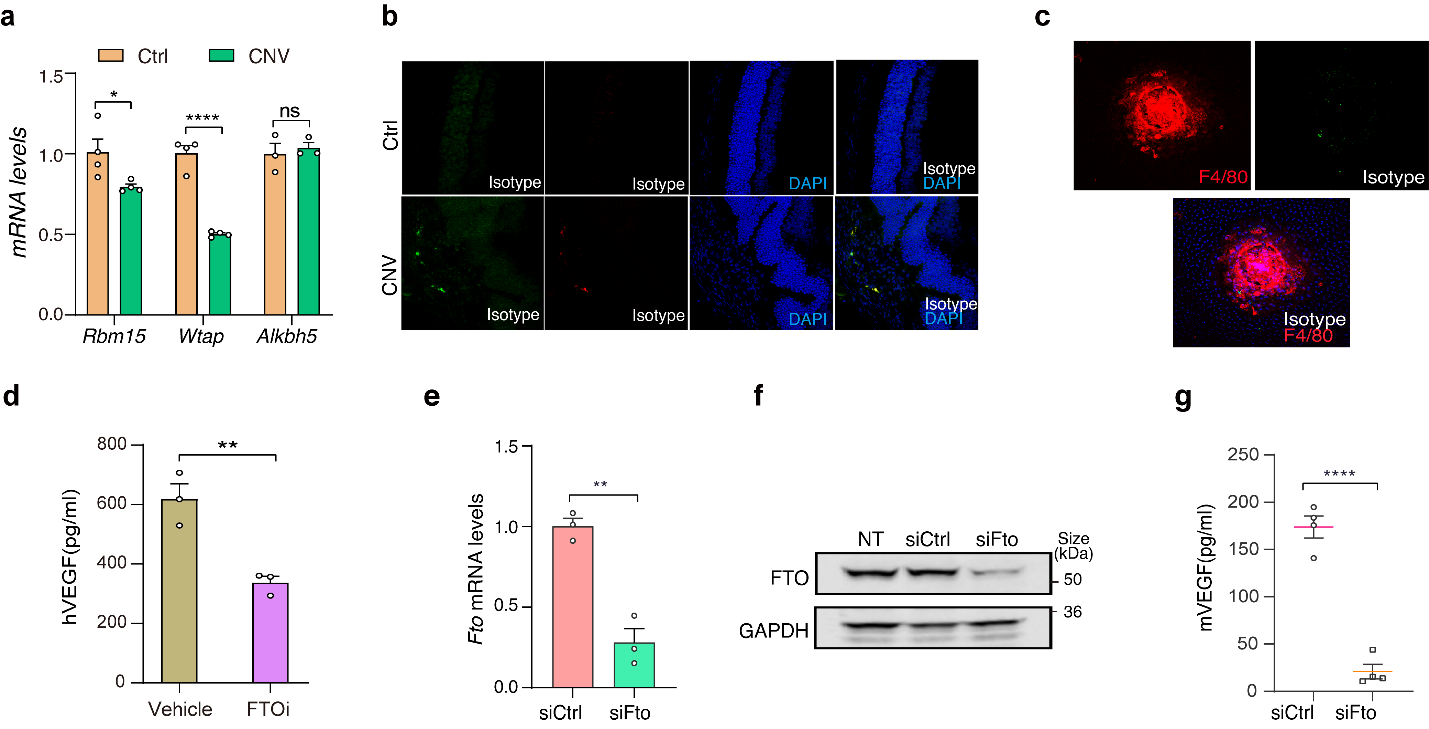


**FTO knockdown suppress VEGF release in mouse macrophage.**

**a.** Quantification of *Alkbh5, Rbm15 and Wtap* in RPE/Choroid tissue of wild-type mice at 3 days after laser injury. Ctrl, control; CNV, choroidal neovascularization. n=3. two-way ANOVA with Sidak’s multiple comparisons. *ns*, not significant. **b, c.** Isotype control of FTO immunofluorescent staining of choroidal angiogenesis in wild-type mice at 3 days after laser injury. Staining of F4/80, the macrophage marker, highlights the CNV lesions. **d.** Measurement of VEGFA release in ARPE-19 cells after treated with 5 µM of FTO inhibitor or vehicle for 24 hours. Error bars represent SEM. n=3. ***p*<0.01. Unpaired two-tailed *t* test. n=3 **e**, **f.** Validation of siRNA knockdown efficiency on BMDM at 24 hours after double FTO siRNA transfection by RT-qPCR (e) and immunoblotting (f) Error bars represent SEM. n=3. ***p*<0.01. Unpaired two-tailed *t* test. NT, non-transfection. **g.** Measurement of VEGFA release in BMDMs at 24 hours after transfection with Control siRNA (*siCtrl*) or *Fto* siRNA (*siFto*).

Figure S2


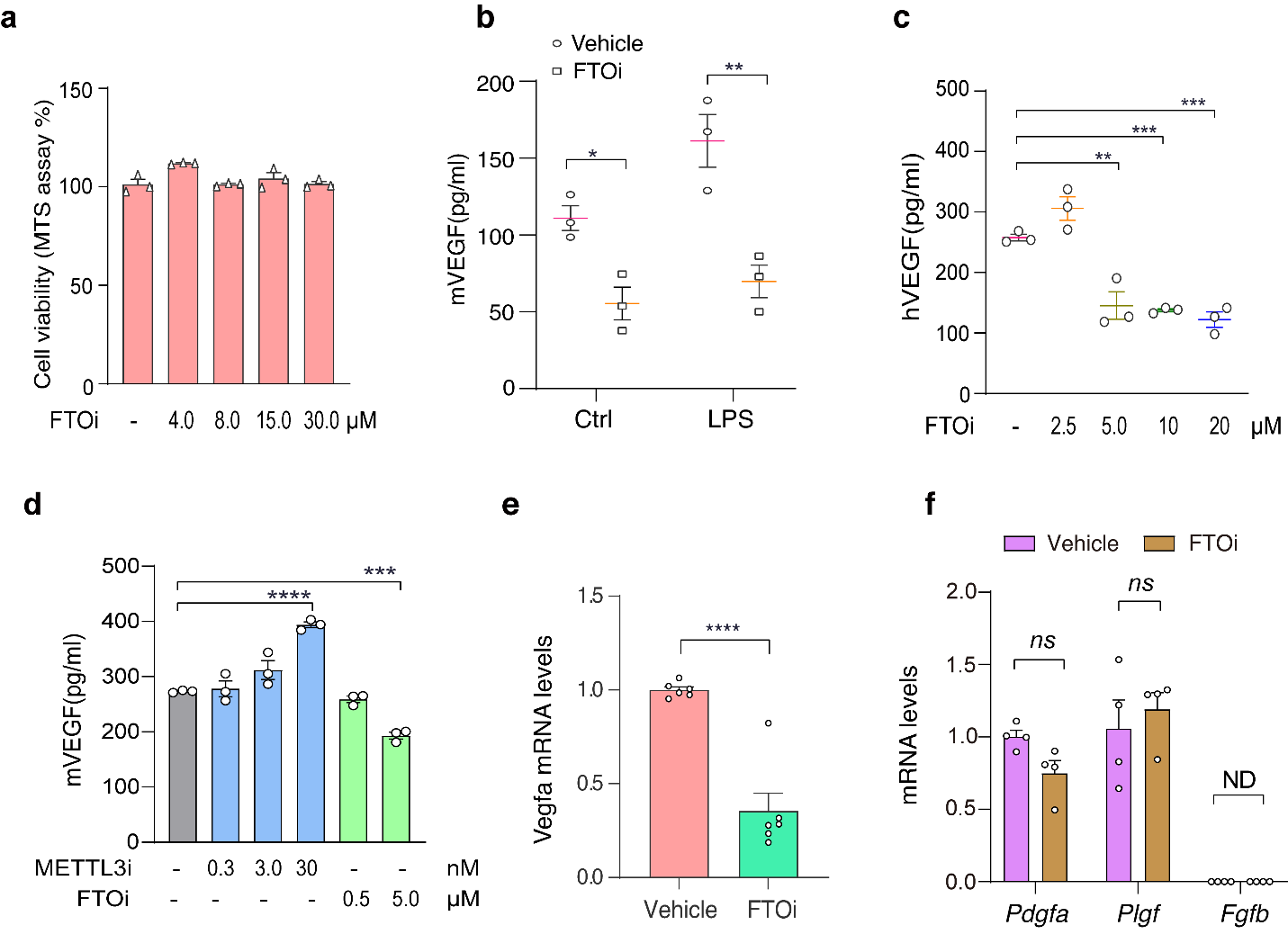


**Pharmacological targeting FTO suppresses VEGF release in mouse macrophage.**

**a.** Cell viability of BMDMs treated with indicated doses of FTO inhibitor for 24 hours, measured by MTS assay. **b.** Measurement of secreted VEGFA in Control (Ctrl) or LPS-primed wild-type BMDMs treated with 5 µM of FTO inhibitor or vehicle for 24 hours. Mean ± SEM for independent experiments. n=3. **p*<0.05; ***p*<0.01. Two-way ANOVA with Sidak’s multiple comparisons. **c.** Measurement of secreted VEGFA in human monocytes (THP-1) treated with indicated doses of FTO inhibitor for 24 hours. Mean ± SEM for independent experiments. n=3. ***p*<0.01; ****p*<0.001. Two-way ANOVA with Sidak’s multiple comparisons. **d.** Measurement of secreted VEGFA in BMDMs treated with an inhibitor targeting the m6A methyltransferase, METTL3 (METTL3i) or an inhibitor targeting FTO (FTOi) for 24 hours. Mean ± SEM for independent experiments. n=3. ****p*<0.001; *****p*<0.0001. Two-way ANOVA with Sidak’s multiple comparisons. **e.** Quantification of *Vegfa* mRNA levels in BMDMs pretreated with FTO inhibitor for 24 hours before adding Actinomycin D. Error bars represent SEM. n=3. *****p*<0.0001. Unpaired two-tailed *t* test. **f.** Quantification of *Pdgfa, Plgf* and *Fgfb* mRNA levels in BMDM pretreated with FTO inhibitor for 24 hours. Error bars represent SEM. n=3. ns, not significant, ND, not detectable.

Figure S3


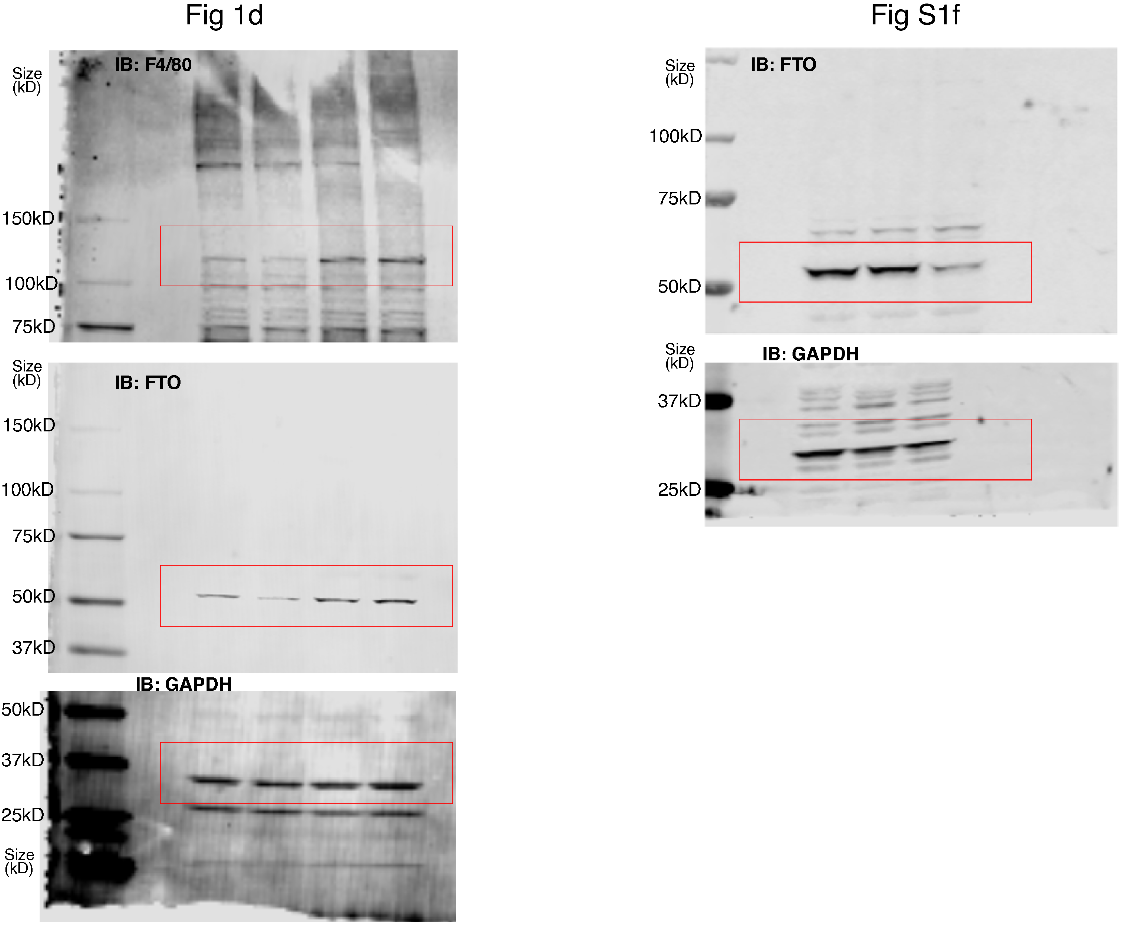


**Uncropped images of western blots for Fig 1d, and Supplementary Fig S1f.**

**Supplementary References**

1 Nozaki, M. *et al.* Drusen complement components C3a and C5a promote choroidal neovascularization. *Proc Natl Acad Sci U S A* **103**, 2328-2333, (2006).

2 Sakurai, E., Anand, A., Ambati, B. K., van Rooijen, N. & Ambati, J. Macrophage depletion inhibits experimental choroidal neovascularization. *Invest Ophthalmol Vis Sci* **44**, 3578-3585, (2003).

3 Kleinman, M. E. *et al.* Sequence- and target-independent angiogenesis suppression by siRNA via TLR3. *Nature* **452**, 591-597, (2008).

4 Weischenfeldt, J. & Porse, B. Bone Marrow-Derived Macrophages (BMM): Isolation and Applications. *CSH Protoc* **2008**, pdb prot5080, (2008).

5 Campos, M., Amaral, J., Becerra, S. P. & Fariss, R. N. A novel imaging technique for experimental choroidal neovascularization. *Invest Ophthalmol Vis Sci* **47**, 5163-5170, (2006).

6 Ratnadiwakara, M. & Anko, M. L. mRNA Stability Assay Using transcription inhibition by Actinomycin D in Mouse Pluripotent Stem Cells. *Bio Protoc* **8**, e3072, (2018).
