# Supplementary figures and images for: Targeting the m6A mRNA demethylase FTO suppresses vascular endothelial growth factor release and choroidal neovascularization
Source: Signal Transduct Target Ther. 2023 Feb 20;8:72. doi: 10.1038/s41392-022-01277-4 (PMC9939410; doi:10.1038/s41392-022-01277-4)

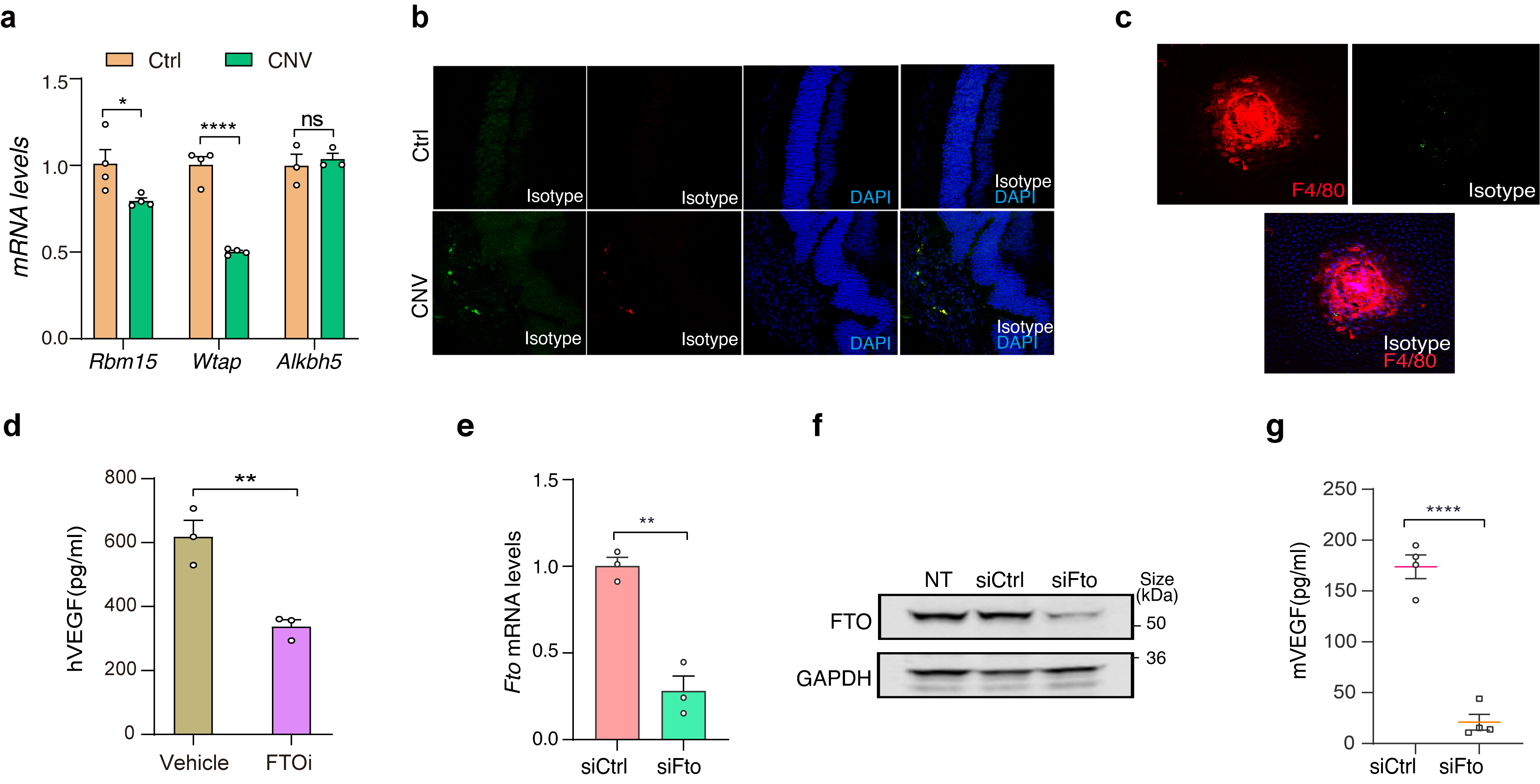

Supplement: Supplementary file 2 — Supplemental Figure 1 [file 41392_2022_1277_MOESM2_ESM.tif]

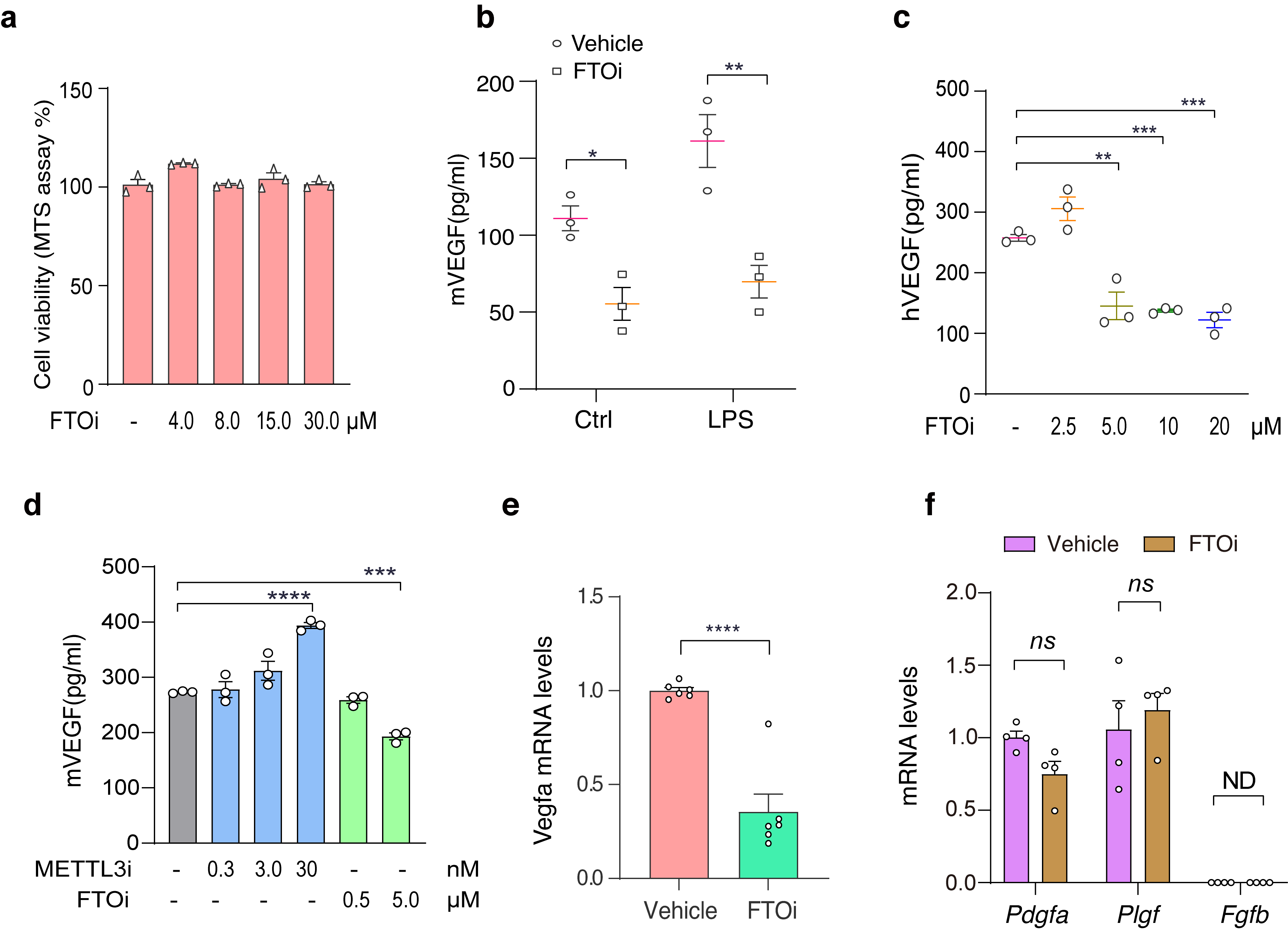

Supplement: Supplementary file 3 — Supplemental Figure 2 [file 41392_2022_1277_MOESM3_ESM.tif]

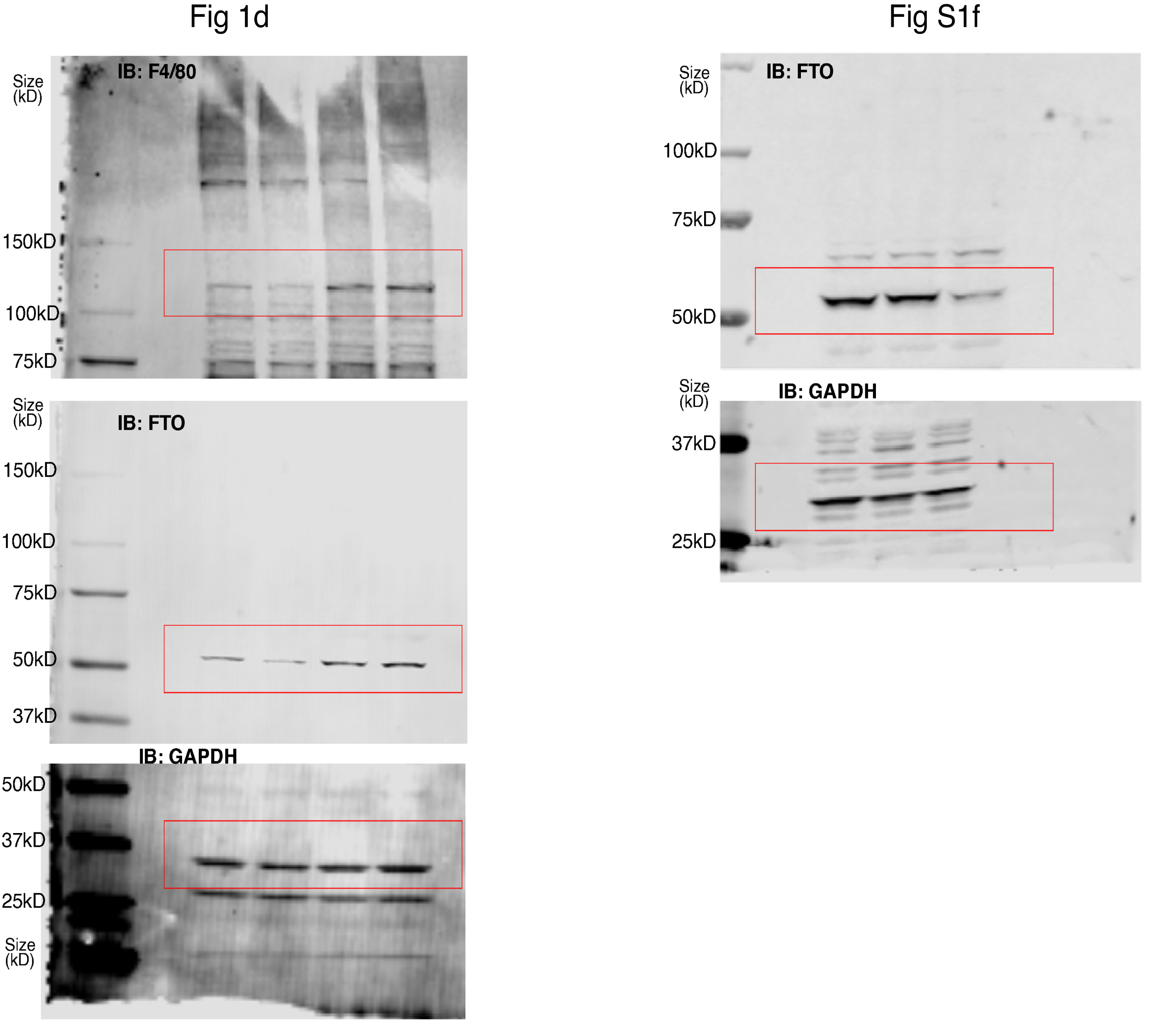

Supplement: Supplementary file 4 — Supplemental Figure 3 [file 41392_2022_1277_MOESM4_ESM.tif]
